# Laparoscopic management of recurrent ureteropelvic junction obstruction following pyeloplasty: a single surgical team experience with 38 cases

**DOI:** 10.1590/S1677-5538.IBJU.2016.0198

**Published:** 2017

**Authors:** Francesco Chiancone, Maurizio Fedelini, Luigi Pucci, Clemente Meccariello, Paolo Fedelini

**Affiliations:** 1 Urologic Clinic, AORN Cardarelli Hospital, Naples, Italy

**Keywords:** Laparoscopy, Hydronephrosis, Recurrence, Kidney Pelvis

## Abstract

**Purpose:**

To describe and analyze our experience with Anderson-Hynes transperitoneal laparoscopic pyeloplasty (LP) in the treatment of recurrent ureteropelvic junction obstruction (UPJO).

**Materials and methods:**

38 consecutive patients who underwent transperitoneal laparoscopic redo-pyeloplasty between January 2007 and January 2015 at our department were included in the analysis. 36 patients were previously treated with dismembered pyeloplasty and 2 patients underwent a retrograde endopyelotomy. All patients were symptomatic and all patients had a T1/2>20 minutes at pre-operative DTPA (diethylene-triamine-pentaacetate) renal scan. All data were collected in a prospectively maintained database and retrospectively analyzed. Intraoperative and postoperative complications have been reported according to the Satava and the Clavien-Dindo system. Treatment success was evaluated by a 12 month-postoperative renal scan. Total success was defined as T1/2≤10 minutes while relative success was defined as T1/2between 10 to 20 minutes. Post-operative hydronephrosis and flank pain were also evaluated.

**Results:**

Mean operating time was 103.16±30 minutes. The mean blood loss was 122.37±73.25mL. The mean postoperative hospital stay was 4.47±0.86 days. No intraoperative complications occurred. 6 out of 38 patients (15.8%) experienced postoperative complications. The success rate was 97.4% for flank pain and 97.4% for hydronephrosis. Post-operative renal scan showed radiological failure in one out of 38 (2.6%) patients, relative success in 2 out of 38 (5.3%) patients and total success in 35 out of 38 (92.1%) of patients.

**Conclusion:**

Laparoscopic redo-pyeloplasty is a feasible procedure for the treatment of recurrent ureteropelvic junction obstruction (UPJO), with a low rate of post-operative complications and a high success rate in high laparoscopic volume centers.

## INTRODUCTION

The failure of laparoscopic pyeloplasty can be early or late. In the early failure, the manifestation is often with pain, fever or a worsening of hydronephrosis after removing the ureteral stent. Routine follow-up after a pyeloplasty consists of ultrasonography, intravenous urography, computed tomography and renal scan. Criteria of success are radiologic and/or clinical improvement or resolution of obstruction. Renal scintigraphic criteria seems to be the best criteria to take into consideration a successful pyeloplasty. About 75% of patients who experienced obstruction after a laparoscopic pyeloplasty based on scintigraphic criteria were asymptomatic, showing a bad correlation between obstruction and symptoms (1). Moreover, the patients can have a nonobstructive significant hydronephrosis and a residual atonic pelvis after pyeloplasty. In that case they can exhibit delayed t1/2 in the “indeterminate” or “obstructed” range (2).

Late failure can also occur two or more years after surgery (3). There are only a few reports of laparoscopic management of recurrent UPJO.

The largest series concerning transperitoneal laparoscopic redo-pyeloplasty have a successful rate of 83% (4) and 88% (5) (involving respectively 36 and 17 patients). The aim of this study was to describe and analyze our experience with Anderson-Hynes transperitoneal laparoscopic pyeloplasty (LP) in the treatment of recurrent ureteropelvic junction obstruction (UPJO).

## MATERIALS AND METHODS

We enrolled thirty-eight consecutive patients who underwent laparoscopic redo-pyeloplasty between January 2007 and January 2015 at our department.

All patients were symptomatic and experienced several episodes of pain. The visual analog scale (VAS) was used to assess pain intensity at the time of colic.

**Figure 1:**
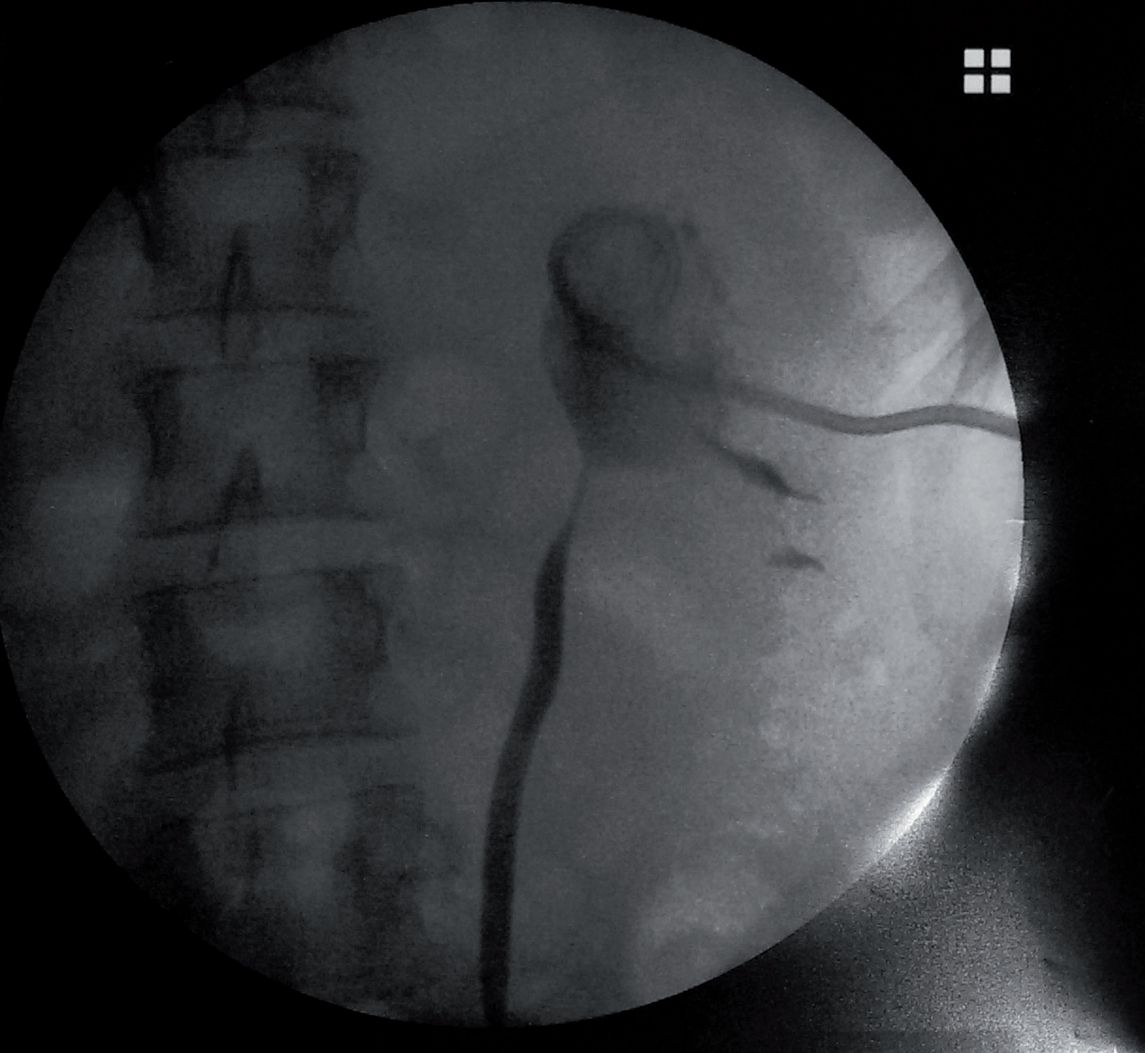
Shows a recurrent UPJO (ureteropelvic junction obstruction) with a percutaneous nephrostomic tube.

All patients were studied preoperatively with renal ultrasonography (US), renal scan, and intravenous urography (IVU) or a CT scan (CT). In all patients, diagnostic tools showed severe hydronephrosis. All patients had immediately a temporary urinary derivation. 28 patients who had not fever (73.7%) underwent an ureteral stent insertion, while 10 patients (26.3%) who had fever underwent a placement of percutaneous nephrostomic tube (Figure-1).

In all cases a transperitoneal pyeloplasty using the Anderson-Hynes technique was performed, by a single surgical laparoscopic team (6).

Intraoperative and postoperative complications have been classified and reported according to Satava (7) and the Clavien-Dindo system (8).

Treatment success was evaluated by 12 months postoperative DTPA (diethylene-triamine-pentaacetate) renal scan, hydronephrosis and flank pain. Total success was defined as T1/2≤10 minutes while relative success was defined as T1/2 between 10 to 20 minutes (9). All patients underwent a periodical clinical and radiological follow-up. All data were collected in a prospectively maintained database and retrospectively analyzed. Descriptive statistics of categorical variables focused on frequencies and proportions. Means and standard deviation were reported for continuously coded variables.

## SURGICAL PROCEDURE

All procedures were performed in lateral decubitus after placement of the ureteral catheter in retrograde fashion and a retrograde ureterography was performed. An open Hasson approach was initially performed using a Hasson cannula. A 0º telescopic and 2 multi-disposal metal trocars (1 x 10-11mm, 1 x 5mm) were used. Dissection was performed by using monopolar scissors and bipolar forceps. The proximal ureter was spatuled with a lateral incision after resection and removal of the stenotic ureteropelvic junction. When we encountered a ventrally crossing vessel we opted to transpose dorsally to the UPJ. The anastomosis was performed using a running 5-0 absorbable suture. A double-J stent was routinely inserted in retrograde fashion but in male patients this step was completed at the end of the laparoscopic intervention under fluoroscopic and cistoscopic control (6).

## RESULTS

**Figure 2:**
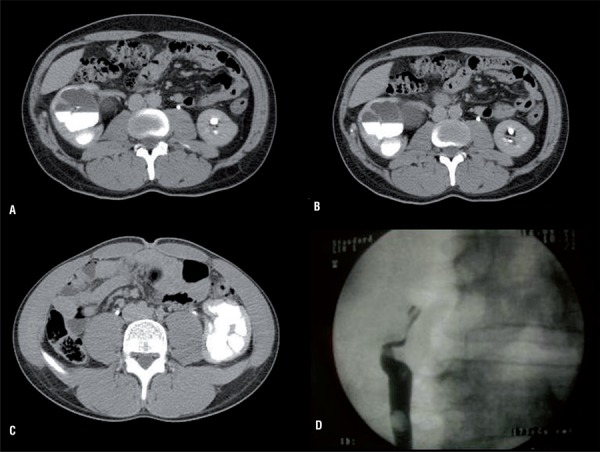
a, b) shows a recurrent UPJO (ureteropelvic junction obstruction) due to an abnormal crossing vessel and (c, d) a recurrent UPJO due to a twisted anastomosis.

Table-1 depicts patient’s demographics and baseline characteristics. The mean age was 26.6±6.5. Body mass index (BMI) was 25.6±2.5. Out of the 38 cases, 16 (42.1%) were males and 22 (57.9%) were females. 12 out of 38 (31.6%) patients performed their first laparoscopic transperitoneal pyeloplasty at our hospital. In two patients, a kidney stone was associated to UPJO. 24 out of 38 (63.2%) patients performed their first pyeloplasty at other hospitals (14 out of 24 procedures were performed using the retroperitoneal open technique and 10 out of 24 using the laparoscopic transperitoneal technique). Two patients (5.3%) underwent a retrograde endopyelotomy at other hospitals. In 28 cases surgical indication was recurrence of UPJO, in 4 cases it was recurrence of UPJO associated with the presence of an abnormal crossing vessel, in 2 cases it was a twisted anastomosis and in 4 cases it was a recurrence of UPJO associated with an incorrect angle of the anastomosis (Figure-2). In 20 (52.6%) cases UPJO was on right side while in 18 (47.4%) cases it was on the left side.

**Table 1:** Demographics and baseline characteristics of the 38 patients.

Variable	Value
	Mean±SD
	
Age at surgery (years)	26.6±6.5
BMI (kg/m^2^)	25.6±2.5
	
	**N±(%)**
	
Males	16 (42.1%)
Females	22 (57.9%)
Right side	20 (52.6%)
Left side	18 (47.4%)
Symptomatic(pain)	38 (100%)
Fever	10 (26.3%)
**Preoperative renal scan:**	
T1/2>20 minutes	38 (100%)
**First treatment:**	
Laparoscopic transperitoneal pyeloplasty (our hospital)	12 (31.6%)
Laparoscopic transperitoneal pyeloplasty (other hospitals)	10 (26.3%)
Retroperitoneal open pyeloplasty (other hospitals)	14 (36.8%)
Retrograde endopyelotomy (other hospitals)	2 (5.3%)
	
**Surgical indication:**	
Recurrence of UPJO	28 (73.7%)
Recurrence of UPJO and abnormal crossing vessel	4 (10.5%)
Twisted anastomosis	2 (5.3%)
Recurrence of UPJO and incorrect angle of the anastomosis	4 (10.5%)
	
	**Mean±SD**
	
Mean stricture length (cm)	0.99±0.45

Mean stricture length was 0.99±0.45cm (range, 0.2-2.2cm) on IVU or retrograde pyelography. All patients were symptomatic and reported at least one episode of severe flank pain (VAS score 7-10) (10). All patients had a T1/2>20 minutes at pre-operative renal scan. 10 out of 38 (26.3%) cases reported at least one episode of fever.

Mean operating time was 103.16±30 minutes and all procedures were fully performed laparoscopically. The mean blood loss was 122.37±73.25 milliliters and no blood transfusions were necessary. The mean postoperative hospital stay was 4.47±0.86 days. Foley catheter was removed postoperatively after 2.9±0.75 days and peritoneal drainage tube was removed if its output didn’t increase within 24 hours after catheter removal. The anomalous crossing vessel was transposed to ureteropelvic junction UPJ dorsally due to evident obstruction in all four patients. The double-J stent was removed after 29.9±5.4 days postoperatively. No intraoperative complications occurred according the Satava system.

Table-2 reports post-operative complications according Clavien-Dindo classification and their management. 6 out of 38 patients (15.8%) experienced postoperative complications: hematuria (2 patients; 5.3%; Clavien-Dindo I), postoperative pain that required analgesics (2 patients; 5.3%; Clavien-Dindo I), urinary tract infection (1 patient; 2.6%; Clavien-Dindo II), urine leakage (1 patient; 2.6%; Clavien-Dindo IIIa).

**Table 2:** Post-operative complications according to Clavien-Dindo classification and their management.

	Grade	n(%)	Management
			
Hematuria	I	2/38 (5.3%)	delayed catheter removal
Postoperative pain	I	2/38 (5.3%)	analgesics
			
Urinary tract infection	II	1/38 (2.6%)	prolonged antibiotics
			
Urine leakage	IIIa	1/38 (2.6%)	percutaneous nephrostomy catheter placement and late removal of double-J stenting
			
Overall		6/38 (15.8%)	

The success rate was 97.4% (36 out of 38 patients) for flank pain using the VAS, and 97.4% (36 out of 38 patients) for hydronephrosis. Post-operative DTPA renal scan at 12 months showed radiological failure in 1 out of 38 (2.6%) patients, relative success in 2 out of 38 (5.3%) patients and total success in 35 out of 38 (92.1%) of patients. The radiologic failure, associated to flank pain and hydronephrosis, occurred in the patient that experienced the urine leakage. The patient underwent a laparoscopic pyeloplasty at our hospital for the third time with relative success at post-operative DTPA renal scan. The mean clinical and radiological follow-up was of 42.5±24.6 months.

## DISCUSSION

Failure of pyeloplasty can be related to different factors. Even if anatomical features play a role, it is most likely secondary to technical issues. In our series one patient had a failure 15 years postoperatively, although most failures presented within 12 months of follow-up.

To obtain a successful pyeloplasty some basic surgical principles should be observed: scrupulous preservation of the vascularity of ureter and pelvis, performing of a widely patent and watertight anastomosis, and careful tissue handling (11). It is important also to perform a “tension free” anastomosis, an anatomic reconstruction of ureteropelvic junction. Care should be taken to avoid kinking or twisting of anastomosis. In order to avoid a twisted anastomosis it is important to perform a good isolation of the pelvis and of the ureter and to pay attention to the first suture point.

Moreover, each crossing blood vessel should be recognized and in case of evident obstruction should be transposed. Some lower pole vessels could not be recognized as the main cause of UPJO during the first operation. In fact, they could have become adherent to an inflamed renal pelvis and could have inferiorly displaced by a big renal pelvis without an important cause of obstruction (12). Patients with a failed pyeloplasty have often an excessive amount of scaring and peripelvic fibrosis, and this finding could be associated to urinary extravasation, or an excessive tissue reaction to the first surgical procedure (13). In fact, one most delicate surgical step is the insertion of the ureteral stent. If the stent is inserted incorrectly, it will cause intraoperative complications or induce moderate to severe postoperative complications as urinary extravasation or fistulas, which lead to peripelvic fibrosis. The urinary extravasation could have caused the only radiological failure in our series.

Nowadays, several options are used for managing the failed pyeloplasty: antegrade or retrograde laser endopyelotomy, balloon dilation, redo-pyeloplasty and ureterocalicostomy. Open redo-pyeloplasty is associated with best outcomes compared with endopyelotomy (14, 15) and it has been the gold standard for years. With the advent of laparoscopy, laparoscopic redo-pyeloplasty has become a realistic alternative to redo open pyeloplasty, even if this approach is still anecdotal in literature. Although laparoscopic redo pyeloplasty may require a longer operative time to release peripelvic and periureteric fibrosis, hospital stay and postoperative complications were less than open redo pieloplasty (16).

Sundaram et al. reported the largest series of laparoscopic redo-pyeloplasty (36 patients) with a successful rate of 83% (4). Nevertheless, only 3 out of 36 (8.3%) patients underwent a pyeloplasty, while in our study 36 out of 38 (94.7%) patients were previously treated with dismembered pyeloplasty.

In our series the success rate was 97.4% for flank pain and 97.4% for hydronephrosis and total success at 12-month post-operative renal scan was achieved in 35 out of 38 (92.1%) of patients.

Radiological failure rate was 2.6%. It was similar to the failure rate previously described for the treatment of the primary UPJO (6). This can be explained by the fact that all procedures were performed by a well-trained and very experienced laparoscopic surgical team. Laparoscopic redopyeloplasty can be a very challenging procedure because some adjuvant maneuvers may be required to success, like the use of a pelvis flap or ureterocalicostomy (17). In situations where ureteric and renal pelvis repair are not possible ileal interposition or autotransplantation can be also considered. The high rate of success in our series can be related to the short length of the failed stenosis without the need for additional challenging maneuvers. In the most complex cases we need to perform the isolation of all kidney and distal ureter in order to perform a tension free anastomosis and to avoid the twisting of the anastomosis.

Some limitations of the study herein include, firstly, the short follow-up time. Another limitation is that all procedures were performed by a single surgical team with significant expertise in laparoscopic surgery, which may restrict the generalizability of our results to centers with more limited laparoscopic experience. Moreover, this is a retrospective observational non-comparative study.

## CONCLUSIONS

Laparoscopic redo-pyeloplasty is a feasible procedure for the treatment of recurrent ureteropelvic junction obstruction (UPJO), with a low rate of post-operative complications and a high success rate in high laparoscopic volume centers.
